# Female mouse tears contain an anti-aggression pheromone

**DOI:** 10.1038/s41598-020-59293-9

**Published:** 2020-02-13

**Authors:** Rosa Maria Cavaliere, Lucia Silvotti, Riccardo Percudani, Roberto Tirindelli

**Affiliations:** 1 Department of Medicine and Surgery, Neuroscience Unit, University of Parma, Via Volturno, 39, 43125 Parma, Italy; 2 Department of Chemistry, Life Sciences and Environmental Sustainability, University of Parma, Parco Area delle Scienze, 11/A, 43124 Parma, Italy

**Keywords:** Olfactory system, Social behaviour

## Abstract

Tears contain pheromones that trigger specific behavioral responses. In the mouse, male tear fluid is involved in long and short-term effects such as the receptive behavior and pregnancy block in females and the aggression in males. In contrast, pup tears exert an inhibitory effect on male mating behavior, also promoting sexual rejection in females. In the rat, a male lacrimal protein acts as an intraspecific and heterospecific signal enhancing sexual behavior in females and evoking avoidance behavior in mouse. However, behavioral effects of female tears on male behavior have yet to be described. Here, we report that female lacrimal fluid of different mouse strains contains a relatively small and involatile factor that abolishes inter-male aggression switching it into a copulatory behavior. The production of this molecule by the lacrimal glands is not affected by the estrous cycle but it is sensitive to ovariectomy, thus suggesting a control mediated by hormones. Moreover, this lacrimal anti-aggression pheromone modulates the activity of the lateral habenula, a brain area responsible for the valence of the aggressive interactions.

## Introduction

In most animals, the process of personal identification occurs through the emission of signaling molecules, namely pheromones that are secreted in different bodily fluids. In rodents, urine is the most representative source of pheromones and contains factors that convey information about sex, elicit or abolish aggressive or sexual behavior or modulate the hormonal status^1–3^.

In the last fifteen years, however, starting from the observation that the physical approach between animals of the same species occurs via smelling and licking different regions of the body, including the facial and ocular areas, an emerging interest was raised on other biological fluids that could be responsible for the modulation of the socio-sexual behavior^4,5^. This led to the identification of specific lacrimal proteins as a source of pheromones. The extraorbital exocrine peptide 1 (ESP1), for example, is produced by the male mouse and induces lordosis behavior in female mice and represents an auto-stimulatory factor that enhances male aggression by self-exposure in conjunction with urinary stimuli^6,7^. Moreover, the same peptide is also responsible for a long-term pheromonal response, namely the Bruce effect, that refers to the pregnancy block in recently pregnant female mice upon exposure to unfamiliar male cues^8,9^. In contrast, a juvenile lacrimal peptide, ESP22, is expressed in 2- to 3-week-old mice and exerts a strong inhibitory effect on adult male mating behavior, also promoting sexual rejection in females^10,11^. Very recently, another lacrimal protein was identified in the male rat that enhances stopping behavior in females^12^. When detected by male or female mice, the same rat protein acts on the autonomic system by decreasing locomotion, body temperature and heart rate^12^. This finding is noteworthy as it infers the presence of pheromonal cues that operate as intraspecific and interspecific signals, at the same time. As for most urinary pheromones, all these lacrimal peptides bind receptors and activate peripheral and central brain areas that are part of the vomeronasal system^7,10,12,13^. No studies have been reported on the physiological and behavioral effects of female tears in the mouse, a species that bases the socio-sexual interactions on pheromones. In this work, we report the effects of female mouse tear fluid on male behavior. We found that female tears contain a factor that strongly inhibits inter-male aggression. This behavioural response was greatly reduced when the fluid was collected from females which underwent extraorbital lacrimal gland removal or ovariectomy, but it was unaffected by the estrous cycle. Investigation on the nature of this inhibiting factor suggests that it is probably involatile and possesses a relatively small molecular weight. We also observed that female mouse tear fluid activates the lateral habenula, a brain area that controls the valence of the aggressive interactions.

## Results

### Female tears inhibit aggressive behavior in males

**Figure 1:**
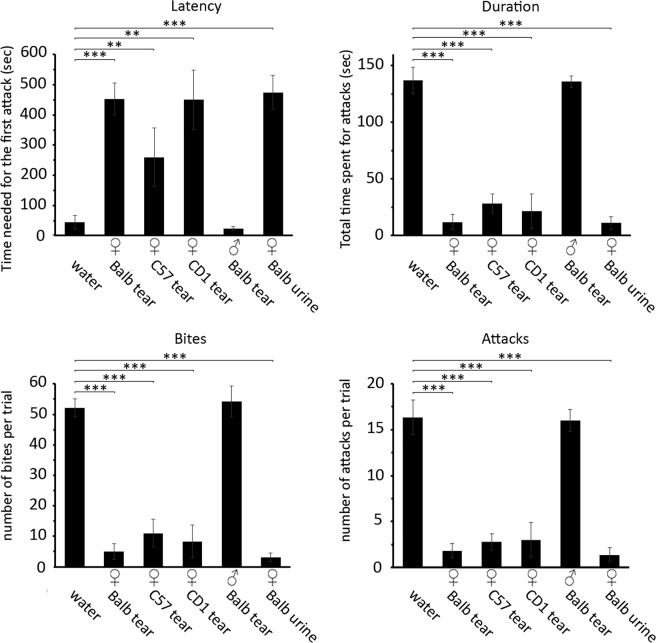
Female tear fluid inhibits aggressive behavior in males. Quantitative analysis of parameters related to aggressive behavior of resident CD1 mice challenged with intruders rubbed with water, urine or tears from different strains. Unpaired *t*-test. **p < 0.01, ***p < 0.001. Mean ± SE; water n = 12, female Balb/c tear n = 18, female C57 tear n = 6, female CD1 tear n = 6, male Balb/c tear n = 6, female Balb/c urine n = 13. The value of each column is reported in Supplementary Dataset S2.

Tear fluids of different mouse strains were tested for their role to modulate inter-male aggression in isolated CD1 mice. Tear fluid was obtained from CD1, Balb/c and C57BL/6 (C57) females and rubbed onto the anal and facial region of male intruders immediately before each experiment. Female tear fluid of all tested strains was able to strongly inhibit aggressive behavior in the resident males (Fig. 1). In the presence of female tear fluid, resident mice significantly decreased the number of attacks (Balb/c, p < 0.001; CD1, p < 0.001; C57, p < 0.001), the duration of the attacks (Balb/c, p < 0.001; CD1, p < 0.001; C57, p < 0.001) and the number of bites (Balb/c, p < 0.001; CD1, p < 0.001; C57, p < 0.001); the latency of the first attack was also significantly increased (Balb/c, p < 0.001; CD1, p = 0.009; C57, p = 0.008). The maximal inhibitory effect was observed when Balb/c female mice were used as tear donors. In contrast, rubbing male tear fluid onto an intruder mouse did not affect the aggression in the resident animal (latency, p = 0.56; duration, p = 0.92; attacks, p = 0.88; bites, p = 0.71) (Fig. 1) (Supplementary Video S1–3). It is worthy of note that male tear fluid did not affect aggression in resident mice also when it was rubbed on castrate intruders, suggesting that male lacrimal secretions do not contain indispensable molecules that allow the identification of the sex of the subject (Supplementary Fig. S1). We also tested plain female urine and confirmed that this fluid was active in reducing the aggressive behavior (latency, p < 0.001; duration, p < 0.001; attacks, p < 0.001; bites, p < 0.001) (Fig. 1). Therefore, although tear collection was performed with every possible precaution (see Methods section), we could not exclude a urinary contamination of the tear fluid, perhaps originating from the fur of the ocular rim. Thus, to dismiss this possibility, we first quantified the creatinine content of the tear fluid. Creatinine is highly concentrated in urine compared to other body fluids, due to water reabsorption by the kidney. Indeed, we found that creatinine concentration in our experimental tear samples of females (0.12 ± 0.02 mg/dL) and males (0.1 ± 0.04 mg/dL) was, on average, more than five hundred times lower than in samples of female pooled urine (58.7 mg ± 4 mg/dL). These are comparable to the concentration measured in salivary fluid (0.15 ± mg/dL) and in plasma (0.17 ± 0.1 mg/dL), thus reflecting the basal concentration of this metabolite in bodily fluids. Then, we diluted urine to bring the concentration of the creatinine to that of the tear fluid (approximately 500 hundred times) and performed an inter-male aggression test with this diluted urinary solution. We found that diluted urine, rubbed onto an intruder mouse, produced a much weaker inhibitory effect on the resident aggressive behaviour compared to that of female tears (latency, p = 0.038; duration, p < 0.001; attacks, p < 0.001; bites, p < 0.001) (Supplementary Fig. S2). Overall, these control experiments indicate that urinary contamination is unlikely to be responsible for the inhibitory effect on aggression induced by female tears. To prove that the aggression-inhibiting factor was indeed originated from tears, we collected tear fluids from Balb/c female mice that underwent a surgical operation for the removal of the extraorbital glands. These glands are known to produce a large proportion of the ocular secretions in the mouse^14^. Tear fluid collected from operated animals, when rubbed onto an intruder mouse, was significantly less effective than the normal fluid to inhibit aggressive behavior of the resident (latency, p = 0.002; duration, p < 0.001; attacks, p < 0.001; bites, p < 0.001) (Supplementary Fig. S3). However, compared to control, tears from operated females retained a weaker inhibitory effect on the duration of fighting, number of attacks, number of bites of the resident behavior (latency, p = 0.195; duration, p = 0.01; attacks, p < 0.001; bites, p < 0.001), suggesting that other lacrimal secretions may be involved in producing the factor responsible for this effect (Supplementary Fig. S3).

### The anti-aggression pheromone is hormonally modulated

**Figure 2:**
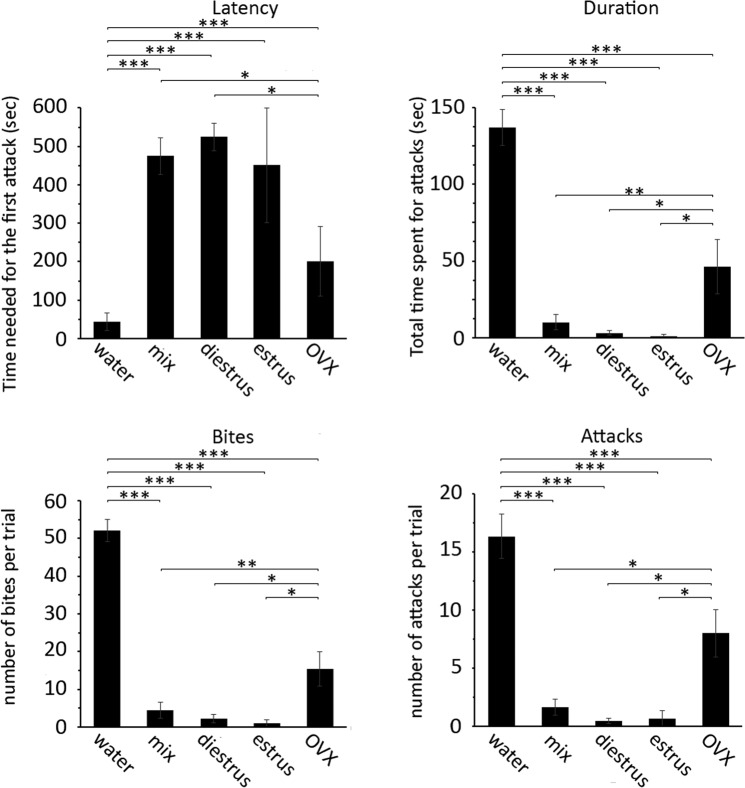
Estrous cycle and ovariectomy differently affect the production of the lacrimal aggression-inhibiting factor. Quantitative analysis indicates that tear fluid from ovariectomized females (OVX) strongly affects male aggression. In contrast, aggression is not modulated by tear fluid obtained from females in different estrous phases. Mix represents pooled estrus and diestrus tears. The values of control column (water) is imported from Fig. 1. Unpaired *t*-test. *p < 0.05, ***p < 0.001. Mean ± SE; n = 5. The value of each column is reported in Supplementary Dataset S2.

Since female mice undergo an estrous cycle lasting approximately four days, the serial eyewashes for tear collection that had been carried out for over two weeks (see Methods), were likely to include tear fluids from all phases of the cycle. Thus, we wondered if the production of the anti-aggression pheromone might be modulated by the hormonal status of females. To scrutinize this issue, tear fluid was specifically collected from females during estrus and diestrus and employed in the inter-male aggression test. Our results suggest that the production of this aggression inhibiting factor is not affected by either diestrus (latency, p < 0.001; duration, p < 0.001; attacks, p < 0.001; bites, p < 0.001) or estrus (latency, p < 0.001; duration, p < 0.001; attacks, p < 0.001; bites, p < 0.001); however, both estrus and diestrus tear fluids displayed a comparable effect on male aggression (latency, p = 0.65; duration, p = 0.62; attacks, p = 0.84; bites, p = 0.67) (Fig. 2). In contrast, tears from ovariectomized females were less effective in inhibiting male aggressive behavior than diestrus tears (latency, p = 0.034; duration, p = 0.04; attacks, p = 0.019; bites, p < 0.042), estrus tears (duration, p = 0.048; attacks, p = 0.037; bites, p = 0.032) or a mix of estrus and diestrus tears (latency, p = 0.032; duration, p = 0.001; attacks, p = 0.021; bites, p < 0.001) (Fig. 2).

### Female tears elicit copulatory behavior between males

**Figure 3:**
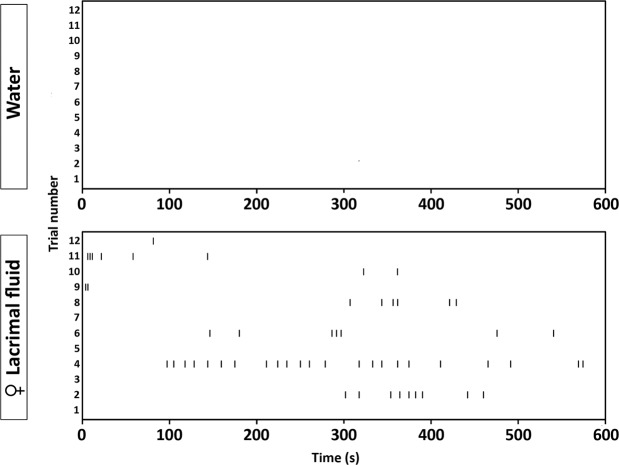
Female tear fluid induces copulatory behavior in resident males. Raster plots depicting individual mounting displays of resident towards intruder males in an inter-male aggression test. Ticks indicate the onset of each mount.

We then asked whether female tears might act as a distracting rather than a sexual stimulus on male aggression. To exclude this possibility, the sexual behavior of resident mice was analyzed during all experimental trials. We found that the majority (8 out of 12) of resident mice exhibited significant mounting displays towards intruders when rubbed with female tear fluid. In contrast, mounting behavior was never observed when intruders were rubbed with water or male tear fluid (Fig. 3). This suggests that the inhibiting effect of female tears on aggression is strongly associated with gender misrecognition.

### Preliminary characterization of the anti-aggression pheromone

**Figure 4:**
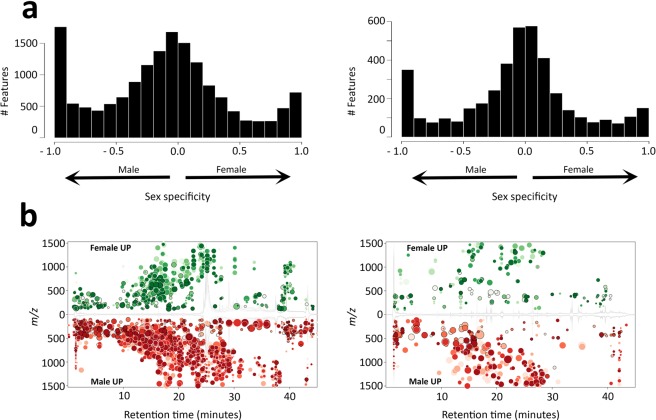
(**a**) Distribution of sex-specificities across components acquired in positive (left panel) and negative (right panel) mode. Results are from three pooled tear samples for each sex as described in the Methods section. Sex-specificity index is calculated as (IF-IM)/(IF + IM) where I is the mean ion intensity of individual components in female (F) and male (M) samples. (**b**) Differential distribution of molecular components in the tear fluid of male and female mice. Only features with a *p*-value < 0.05 and fold change >1.5 are displayed. Upregulated features obtained in positive (left panel) and negative (right panel) mode are shown in green (females *vs* males) or red (males *vs* females). The size of each bubble corresponds to the Log fold change of that feature. The shade of the bubbles corresponds to the magnitude of the *p*-value (the darker the color, the smaller the *p*-value).

As tear fluid is enriched in relatively high molecular weight pheromones (peptides and proteins) that are well-documented to elicit distinct behavioral responses, we wanted to estimate the molecular size of the anti-aggression pheromone. Thus, female tear fluid was dialyzed against water using a 2 KDa cutoff membrane and the retentate and permeate solutions were tested for their effect on male aggression. Our results show that both these solutions, when re-concentrated at the original working volume, were effective in inhibiting aggression (retentate: latency, p = 0.06; duration, p < 0.001; attacks, p = 0.007; bites, p < 0.001; permeate: latency, p < 0.001; duration, p < 0.001; attacks, p < 0.001; bites, p < 0.001); however, compared to the retentate solution, the permeate solution was significantly more active (latency, p = 0.045; duration, p = 0.01; attacks, p = 0.034; bites, p = 0.008) (Supplementary Fig. S4). Thus, the anti-aggression pheromone of female tears is likely to have a relatively small molecular radius. Moreover, given that tear fluid obtained from eyewashes was always extensively lyophilized before resuspension in the working volume (see Methods section), it is plausible that this factor possesses a high boiling point and, therefore, is rather involatile. To get insights on the sex-specific composition of low molecular weight components in mouse tears we performed metabolomic profiling of male and female tear samples. Three biological replicates of pooled tear samples were analyzed by high-resolution liquid chromatography mass spectroscopy (LC-MS) both in positive and negative mode in the range 100–1500 *m/z* (Fig. 4 and Supplementary Fig. S5). Differential analysis of male and female features showed that the majority of components had a similar abundance in both sexes. However, a substantial fraction of the components appeared to have a sex specific representation (Fig. 4 and Supplementary Fig. S5). By applying a statistical cutoff of *p* < 0.05 we obtained 2833 and 599 components with differential abundance (fold > 1.5) between sexes in positive and negative mode, respectively (Fig. 4 and Supplementary Dataset S1). Among features significantly more abundant in females than males (747 in positive mode and 199 in negative mode), our list comprises components having *m/z* value corresponding to molecules previously identified in urine and implicated in behavioral responses^15^. These include, two features observed in negative mode with a mean *m/z* of 377.20 and 361.20 matching, within the tolerance of the instrument, with cortigynic and corticosteronic acid. However, the abundance ranking of these two features in female lacrimal samples was much lower (below the fiftieth percentile) than that observed in a control of urine sample (higher than the fifth percentile).

### Female tears modulate the neuronal activity during an aggressive context

**Figure 5:**
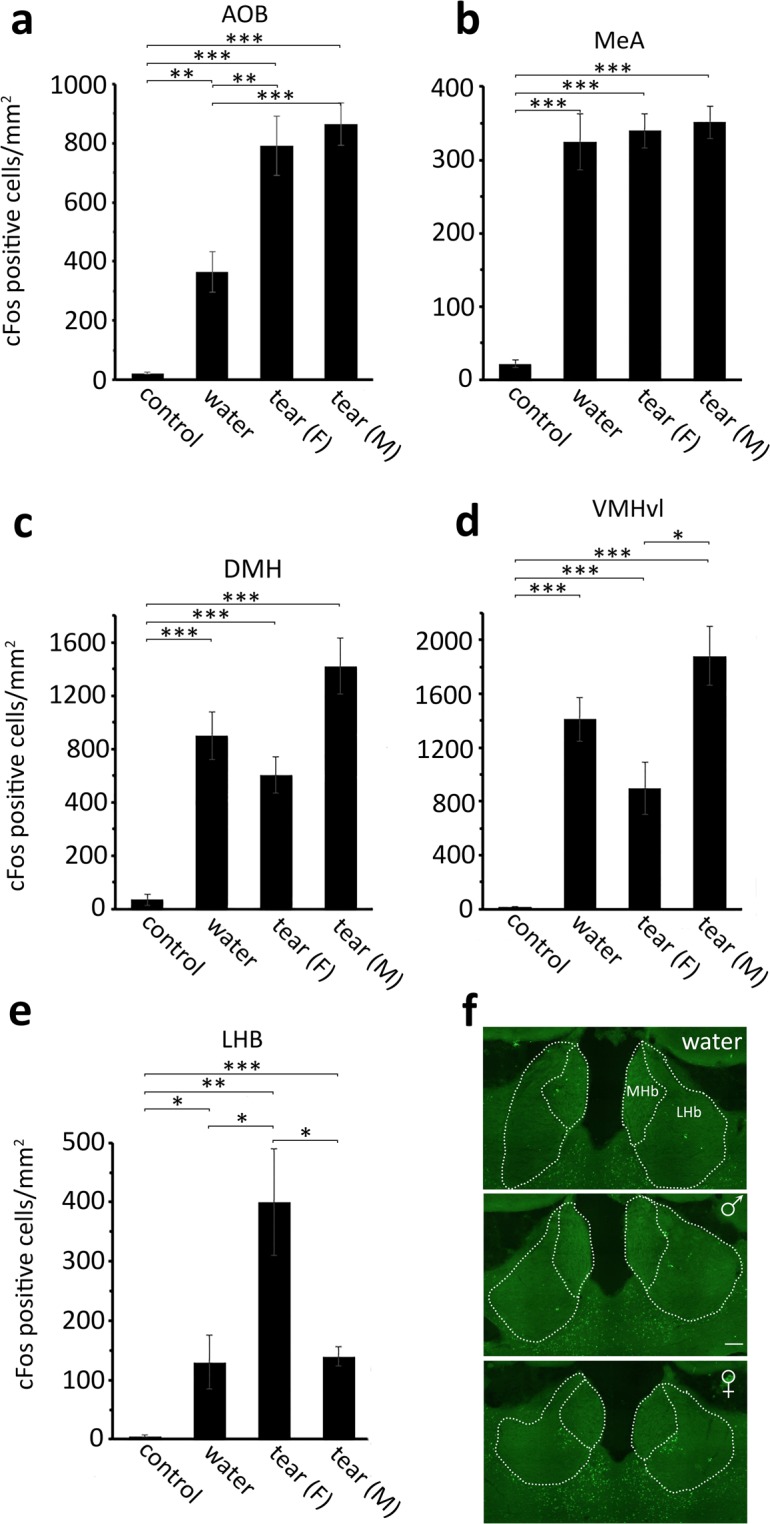
Effect of female tear fluid on c-Fos expression in brain regions controlling aggressive behavior. The number of c-Fos positive cells was measured in (**a**), the accessory olfactory bulb (AOB), (**b**) the medial amygdala (MeA), (**c**) the dorso-medial hypothalamus (DMH), (**d**) the ventrolateral sub-region of the ventromedial hypothalamus (VMHvl) and (**e**) the lateral habenula (LHb) of isolated resident mice (control), resident mice sacrificed after challenging an intruder rubbed with water (water), female (F) or male (M) tear fluids. Unpaired *t*-test. Mean ± SE; n = 6; *p < 0.05, **p < 0.01, ***p < 0.001. The value of each bar is reported in Supplementary Dataset S2. (**e**) Immunostaining of the habenular complex with an antibody against c-Fos in a resident mouse 90′ after exposure to an intruder rubbed with water, female or male tear fluid. (LHb, lateral habenula; MHb, medial habenula). Scale bar = 50 µm.

Aggressive behavior is strongly triggered by the vomeronasal system through the activation of the accessory olfactory bulb (AOB) and medial amygdala (MeA) that sends projections to the ventral-lateral sub-region of the ventromedial hypothalamus (VMHvl) and the dorsal-medial hypothalamus (DMH)^16^. Indeed, in the AOB, we found that when a resident mouse was exposed to an intruder of the same sex, rubbed with water, male or female tear fluid, the number of c-Fos positive cells dramatically increased with respect to control (isolated mouse) (water, p = 0.007; male tears, p < 0.001; female tears, p < 0.001). Interestingly, rubbing male and female lacrimal fluids onto the intruders further augmented the number of c-Fos positive cells in the AOB compared to water painted animals (female tears, p = 0.005; male tears, p < 0.001) (Fig. 5a). Similarly, in the MeA and DMH, when a resident male mouse was exposed to an intruder of the same sex, the number of c-Fos positive cells significantly increased with respect to control (MeA, p < 0.001; DMH, p < 0.001; VMHvl, p < 0.001) (Fig. 5b,c). However, when the intruder mouse was rubbed with female or male tear fluid, we did not observe a further increase, decrease or redistribution of the immunostaining both in MeA (male tears, p = 0.56; female tears, p = 0.75) and DMH (male tears, p = 0.1; female tears, p = 0.24). In the VMHvl, rubbing intruders with female tears induces an evident but still not significant decrease of c-Fos immunoreactivity (p = 0.058) compared to water rubbed intruder; in contrast the activity of VMHvl neurons remained unchanged (p = 0.19) in the presence of male tear fluid. However, c-Fos immunoreactivity was significantly lower in resident mice exposed to female rather than male tears (p = 0.022) (Fig. 5d).

Unexpectedly, we found a remarkable effect of female tears on the neuronal activity of the lateral habenula (LHb) in the resident mouse after an inter-male aggression test (Fig. 5e,f). We observed that aggressive behavior induces c-Fos immunoreactivity in this brain region (water vs control, p = 0.01) that was not modulated by male tear fluid. In contrast, when female tear fluid was rubbed onto intruders during an inter-male aggression test, c-Fos immunoreactivity in the LHb underwent a threefold increase compared to water or male tears (male tears, p = 0.028; water, p = 0.031) (Fig. 5e). Interestingly, we observed that, in contrast to water or male tear fluid, female tears predominantly activated neurons of the medial part of the LHb, in correspondence with the magnicellular and parvicellular subnuclei^17^ (Fig. 5f).

## Discussion

In this study, we have examined the effect of female tear fluid on male aggression during an inter-male aggression test where an intruder mouse was challenged with a resident mouse living isolated in the home cage. We found that female tears of three tested strains (CD1, Balb/c and C57BL/6), when rubbed into the facial and anal region of the intruder mouse almost abolished the aggressive attitude of the resident mouse that was evaluated through different parameters such as latency, number of bites and attacks, and fight duration. In contrast, male tear fluid did not exert any effect on the aggressive behavior of the resident, even when the fluid was rubbed onto a castrate intruder that is usually unable to induce aggression in the resident. Removal of the extraorbital glands dramatically reduced the ability of female tears to inhibit inter-male aggressive behavior suggesting that the aggression-inhibiting factor is abundantly produced by these lacrimal glands. Old studies reported a similar effect elicited by female urine^18–20^. These authors found that ovariectomy abolished the effect whereas estrus and diestrus urines were equally able to inhibit aggression. Indeed, we also confirmed that female urine was as effective as tears in inhibiting aggressive behavior in resident males. Moreover, the production of the tear aggression-inhibiting factor was modulated by ovariectomy but not by the estrus phases as reported for urine. Preliminary attempts to characterize the substance underlying this effect suggest that the aggression-inhibiting factor of tears is barely volatile, water-soluble and possesses a relatively small molecular weight. All this information may suggest that several tissues including exocrine glands produce female specific anti-aggression molecules. Recently, a steroid molecule, termed cortigynic acid, was identified and purified from the urine of adult females^15^. Interestingly, estrus and diestrus urine showed little difference in the content of cortigynic acid whereas ovariectomy results in a significant decrease of the concentration of this molecule in female urine. In behavioral tests, cortigynic acid, when rubbed onto ovariectomized females, increased the investigatory and mounting behavior in males^15^. To identify cortigynic acid and other possible candidates for the role of anti-aggression pheromone, we analyzed female and male tears by mass spectroscopy and estimated the differential abundance of the molecular components in male and female tears. We found that female tear contains a considerable number of molecular components that are significantly more abundant than in males. Amongst these features, cortigynic acid appears to be at least six times more abundant in female than male tear; however, compared to female urine, this molecule is probably present at a thousand-fold dilution in accordance with the comparison of the ion current for this feature in the two biological samples. For this reason and, in the light of our experiments with diluted urine (see Supplementary Fig. S2), we believe that is rather unlikely that the lacrimal cortigynic acid alone is capable of inhibiting aggression in males. Furthermore, cortigynic acid is reportedly a mouse specific molecule and is absent in other rodents^15^, whereas the aggression inhibiting factor appears to be present in urine of different species, suggesting its involvement in a generalized interspecies mechanism for gender recognition^21^. Interestingly, this notion is also supported by the recent observations that pheromones act as both interspecific and intraspecific signaling molecules^12^. In humans, social communication probably occurs through secretions other than urine, for example, sweat whose pheromonal cues are probably detected by the olfactory system^22–25^. It is noteworthy that women’s tears contain a chemosignal that, when smelled by men, decreases the sexual arousal and the salivary testosterone level and attenuates the hypothalamic activation following sexual stimulation^26^. Notably, both testosterone level and the activation of the hypothalamic circuits are known to be tightly linked to human aggressive behavior or to traits that promote aggression^27^. Taken together, the observations of these authors and ours cannot entirely exclude the existence of different pheromonal molecules that are produced in specific body fluids and that exert comparable functions within each species.

In this work, we also tried to trace the central wiring underlying the inhibitory effect of female tears on male aggression. Given the possible involatility of the aggression-inhibiting factor, we envisaged that this molecule accomplished its action through the stimulation/inhibition of the vomeronasal system that is known to mediate inter-male aggression^28,29^. As expected, following the aggression test, we found a strong neuronal activation of c-Fos expression in the accessory olfactory bulb (AOB) and medial amygdala (MeA) that is a pivotal hub overlying sexual communication in rodents; In the AOB, the number of c-Fos positive cells was positively affected by both female and male tear fluid. However, this effect was not recapitulated in the MeA in which water, female and male tears are equally effective in increasing the number of activated neurons. Moreover, we did not find any discernible modification of the spatial map of activation in the MeA upon tear fluid stimulation. Indeed, given that male odors in the aggression context induces the activation of a large subset of MeA neurons and assuming that, in this brain region, different stimuli are known to activate intermingled sets of neurons^30^, it remains plausible that our experimental approach is not sufficiently sensitive to detect very small differences in neuronal activation.

Recently, it was demonstrated that the ventro-lateral part of the ventrolmedial hypothalamus (VMHvl) that receives inhibitory and excitatory inputs from MeA represents the brain locus responsible for the initiation and prosecution of the aggressive behavior in the mouse^31–33^. The agonistic interaction with an intruder increases VMHvl activity in male mice whereas sexual interaction of males with females causes suppression of VMHvl activity^34^. These effects have been reported to involve a specific population of VMHvl neurons that express the estrogen receptor alpha (Esr1). The optogenetic inhibition of Esr1 positive cells results in a dramatic decrease of the initiation and continuation phases of the attack^35^ whereas, more interestingly, the activation of this neuronal subpopulation evoked mounting only, mounting and aggressive, or aggressive only behavior according to the progressive intensity of the stimulus^35^. In this context, we observed a trend (p = 0.058) toward a decrease in the number of c-Fos positive cells in VMHvl of the resident mouse when this is challenged with an intruder rubbed with female but not with male tear fluid or water. It is possible that this trend difference in c-Fos expression could reflect the partial attenuation of the activity of Esr1 positive cells by female cues with the consequence of quenching aggressive performance and switching it to a copulatory behavior in the resident mouse^35^.

Interestingly, in our study, we found that female tear fluid strongly increased c-Fos immunoreactivity in the lateral habenula (LHb), a small epithalamic region that is recognized to be a critical node of the reward circuitry to signal mainly negative (but also positive) valence of a stimulus and promote aversive and emotional states, thus driving motivated behaviors and decision-making^36–38^. Recent optogenetic studies showed that inhibition of GABAergic basal forebrain nuclei input to LHb reduced aggressive behavior during an inter-male aggression test and stimulated conditional place aversion in the resident mouse that was conditioned to associate specific environmental cues with an intruder^39^. Interestingly, LHb neuronal activation is progressively reduced when the intensity of the aggression increases^39^. In the light of these findings, it is possible that the upregulation of c-Fos expression in the LHb of resident mice, smelling an intruder rubbed with female tear fluid, which we observe, is the result of a stimulatory effect exerted by the female fluid on the activation of forebrain structures. This activation would inhibit regions, such as the dorsal raphe nucleus and the ventral tegmental area that, in turn, control the aggressive performance and its valence. Moreover, we found that activated neurons upon female tear fluid stimulation were predominantly concentrated in the medial subnuclei of LHb which are highly enriched in type-2 serotonin receptors (Htr2c)^40^. Interestingly, Htr2c activation inversely correlated with the firing rate in serotoninergic dorsal raphe and the dopaminergic neurons in the ventral tegmental nucleus, both involved in the aggressive behavior^41,42^. Indeed, it will be an interesting goal for future studies to determine the anatomical and physiological connections between the LHb and the brain regions involved in the vomeronasal mediated aggressive responses such as the medial amygdala or the VMHvl.

## Materials and Methods

### Animals

Mice were purchased from Harlan and bred in our animal house. Mice were maintained on a 12 hr dark, 12 hr light cycle. All animal protocols complied with the ethical guidelines for the care and use of laboratory animals issued by the Italian Government and were approved by the Animal Welfare Committee (OPBA, Organizzazione per il Benessere Animale) of the University of Parma (approval ID: 17/14, date: 27/03/14). All methods were carried out in accordance with the relevant guidelines and regulations.

### Fluid collection for behavioral experiments

Each tear fluid batch was usually collected from fifteen isolated three-month-old mice by eyewash with 15 µl of water four times a day for at least two weeks after extensive cleaning of the sub ocular region with a cotton pad soaked in borate solution. Tear fluids collected from ovariectomized females and females deprived of the extraorbital glands were obtained from six operated mice. Diluted tear fluids were pooled, frozen and extensively lyophilized for at least 48 hr to remove the volatile molecules. Dry fluids were then resuspended in water in a tenth of the initial volume. Three Balb/c female tear fluid batches were used for the experiment shown in Fig. 1 and Supplementary Figs. 3–5. Two batches of male tear fluids were used for experiments in Fig. 1 and Supplementary Fig. S2. A single batch of tear fluid was used in the other experiments.

Urine was obtained from each mouse by holding the animal and gently massaging the anogenital region. The urine drops were immediately collected, pooled and frozen at −80 °C.

### Creatinine determination

Before creatinine analysis, samples of pooled urine that were employed in each behavioral experiment were diluted ten times whereas tear and salivary fluids, obtained from ocular and oral washouts, were concentrated thirty times in order to obtain a quantifiable value. Creatinine concentration was determined by the colorimetric methods according to the manufacturer’s instructions (Creatinine Assay Kit, Sigma-Aldrich).

### Inter-male aggression test

Sexually naïve, two-to-three months old CD1 resident male (27–30 gr) mice were isolated for 6 days without change of the bedding. Each resident mouse was subjected to a single test against a CD1 intruder of the same age and weight. Test lasted 10′ and began when the intruder male was placed in the home cage (22 × 16 × 14 cm) of the resident mouse.

All observations were recorded in the same room of the animal house where mice lived, at the same hour of the day (5 pm.), under standard indoor lighting, using a digital camera located laterally to a clear Plexiglass cage. The biological fluids (or water) were applied with a small brush onto the facial and anal regions of the intruder mice immediately before the introduction in the resident cage. Approximately 20–30 µl of fluid was deposited on each mouse according to differential weight of the brush before and after fluid administration. Recorded videos were analyzed and scored by a blinded experimenter using the Observer software to evaluate (1) the latency to initiate the first attack, (2) the number of aggressive attacks, (3) the number of bites, (4) the cumulative time spent for attacks and (5) the socio-sexual behavior.

The aggressive attack was defined by beginning of aggression with continuous positioning of the resident mouse toward the intruder. Each aggression was considered complete when the resident mouse stood apart from the intruder for longer than 3''.

When castrated CD1 males were employed to evaluate the aggressive behavior of the resident (see supplementary Fig. S1), the inter-male aggression test was performed on sexually naïve isolated resident mice that were exposed to an intact intruder to evaluate the basal aggressive attitude (day 1) and, then, for three consecutive days to the same castrate mouse to stabilize the reduction of the aggressive behavior (days 2–4). Finally, in the fifth day, resident was challenged with the same castrate mouse rubbed with male Balb/c tear fluid to observe any modification of the aggressive performances.

### Surgery

Removal of the extraorbital glands and ovariectomy was performed as previously described^43^. For ovariectomy, three-week-old Balb/c female mice were anesthetized with a 0.5 ml PBS solution containing xylazine (0.1 mg/gr weight) and ketamine (0.25 mg/gr weight). Mice were placed ventrally and the dorsal surgical area was shaved and disinfected. A short dorsal midline skin and abdominal muscle incision was made halfway between the last dorsal rib and the base of the tail. The ovary and the oviduct were exteriorized. A sterile ligature was placed around the oviduct and the ovary was removed. The surgical plan was then closed with silk sutures. For lacrimal gland surgery, Balb/c male mice were anesthetized as above described. The extraorbital lacrimal gland in the mouse is located rostrally to the parotid gland and lies on the masseter muscle, draining fluid into the conjunctival sac. A skin incision was made between the lateral commissure of the eye and ear. After dissection of the subcutaneous tissue, the lacrimal gland was identified and exposed. By using forceps, the gland was easily separated from the parotid gland and exteriorized. The lacrimal duct was identified and ligated with non-reabsorbable suture and the gland was removed. Animals were left to recover for two weeks during which the eyes were lubricated and maintained aseptic with eye drops containing antibiotics and Celluvisc^©^.

### Brain tissue preparation and immunohistochemistry

Ninety minutes after the inter-male aggression test, resident mice were deeply anesthetized with a mixture of xylazine (1 mg/gr weight) and ketamine (2.5 mg/gr weight) and transcardiacally perfused with a solution of 4% phosphate-buffered paraformaldehyde for 20′ at room temperature followed by a 30′ perfusion with a 30% sucrose solution in PBS. Subsequently, brain was then dissected and included in OCT embedding solution (CellPath, UK) and frozen in liquid nitrogen-cooled pentane.

Cryostat-cut sections (50 μm) were blocked in 1% albumin, 0.3% Triton X-100 for 20 min and incubated with goat anti-c-Fos antibody (Santa Cruz, 1:100 dilution) in the same blocking solution for 40 hrs. at 4 °C. Slices were then washed in PBS three times, incubated with an anti-goat IgG conjugated with Alexa488 (1:350) (Invitrogen) for 2 hrs at room temperature. Sections were then coverslipped with a solution of PBS and glycerol (1:10). Fluorescent images were obtained using a Zeiss fluorescent microscope (Carl Zeiss, Jena, Germany) at 20X magnification. Identification of the brain areas and nomenclature were according to the Mouse Brain Atlas of Franklin and Paxinos^44^. Images with brain areas of interest were taken and collaged with the Nis-element software (Nikon). All sections of the brain areas of interest in each experimental animal were considered for analysis of c-Fos immunoreactivity. The total number of immunostained nuclei was calculated and normalized to mm^2^ brain area with Photoshop CC-2018 software, to obtain the density of c-Fos positive nuclei of each area, in each animal.

### Fluid profiles by high resolution mass spectrometry

LC-MS profiles of tear fluid and urine were acquired with a LTQ Orbitrap XL, Thermo Fisher Scientific (Waltham, Massachusetts, USA) interfaced with a Dionex Ultimate3000 HPLC, equipped with a XB-C18, 15 cm × 2.1 mm column (Phenomenex). Each run lasted 38 min. using two eluting solutions, 0.2% formic acid in H_2_O, (eluent A), and 0.2% formic acid in pure acetonitrile (eluent B), at a flow rate of 0.2 ml/min. The eluting steps were: 0–5 min, 95% A; 6–31 min from 95% A to 50% A, 32–33 min from 50% A to 5% A; 34–37 min at 5% A; 38 min from 5% to 95% A. Instrument parameters for LTQ Orbitrap were: injection volume 5 μl, spray voltage, 3.5 kV; capillary voltage, −35 V; source temperature, 275 °C; tube lens, −222 V. scan range, 100–1500 *m*/*z*.

Male and female tear samples were separately employed for LC-MS analysis. Each biological replicate consisted of tear fluid collected over 10 days from 10 adult subjects (two to three month old), living in a single cage. Before analysis, each sample was tested for its biological activity. For comparison, the same analysis was performed on pooled urine samples collected over 10 days from the same animals.

For the differential analysis of female and male tear samples, raw data files produced by the LTQ Orbitrap experiments were converted into centroided mzML files using the msconvert program of Proteowizard (http://proteowizard.sourceforge.net) with the option “peakPicking true”. Pairwise comparison of male and female group samples was performed on three biological replicates for each group with the XCMS_online^45^ software at https://xcmsonline.scripps.edu. The analysis was conducted using the Orbitrap parameters with the following modifications: ppm = 5; peakwidth = 10.50; prefilter intensity = 1000; minsamp = 3. Statistics for the sex-specific abundance of individual components were based on the unpaired parametric t-test. Visual maps of MS experiments were constructed from centroided mzML files using the R packages mzR and Msnbase.

### Statistical analysis

For behavioral experiments, randomization was not used and no statistical methods were employed to determine sample size. The comparison of aggressive behavior between groups of CD1 resident mice exposed to intruders treated with different solutions was analyzed. Experimenters were blind to the group distribution during analysis of the data. Data are shown as column charts or points and bars indicate means ± SE. For all the behavioral and immunohistochemical analyses, except from the experiments shown in Supplementary Fig. S1 in which paired t-test was used using SPSS 24.0 Statistics, the statistical significance was obtained by independent samples t-test (unpaired t-test applied to every pairwise combination of conditions) with Levene’s test for equality of variance using SPSS 24.0 Statistics. For all the experiments, p-value < 0.05 was considered statistically significant. The number of animals employed in each experiment was indicated in the captions of figures.

## Supplementary information

Supplementary Figures.

Supplementary Dataset S1.

Supplementary Dataset S2.

Supplementary Video S1.

Supplementary Video S2.

Supplementary Video S3.

## Data Availability

The datasets generated and analyzed during the current study are available from the corresponding author and/or from the Mendeley Data repository (10.17632/bczrv2trtc.1).
