# Author Correction: Female mouse tears contain an anti-aggression pheromone

**DOI:** 10.1038/s41598-020-76734-7

**Published:** 2020-11-10

**Authors:** Rosa Maria Cavaliere, Lucia Silvotti, Riccardo Percudani, Roberto Tirindelli

**Affiliations:** 1 Department of Medicine and Surgery, Neuroscience Unit, University of Parma, Via Volturno, 39, 43125 Parma, Italy; 2 Department of Chemistry, Life Sciences and Environmental Sustainability, University of Parma, Parco Area delle Scienze, 11/A, 43124 Parma, Italy

Correction to: *Scientific Reports* https://doi.org/10.1038/s41598-020-59293-9, published online 13 February 2020.

The Supplementary Information published with this Article contains errors.

The footage contained in Supplementary Video S2 is a duplicate of Supplementary Video S1, which shows “Intermale aggression test: intruder mouse swabbed with water” rather than “Intermale aggression test: intruder mouse swabbed with male tears”.

The correct Supplementary Video S2 file is linked to this correction notice.

## Supplementary information

Supplementary Video S2.

