# Supplementary material for: Female mouse tears contain an anti-aggression pheromone
Source: Sci Rep. 2020 Feb 13;10:2510. doi: 10.1038/s41598-020-59293-9 (PMC7018997; doi:10.1038/s41598-020-59293-9)
Supplement: Supplementary file 1 — Supplementary Figures. [file 41598_2020_59293_MOESM1_ESM.pdf]

Female mouse tears contain an anti-aggression pheromone

Rosa Maria Cavaliere[1], Lucia Silvotti[1], Riccardo Percudani[2] and Roberto Tirindelli[1]*

[1]Department of Medicine and Surgery, Neuroscience Unit, University of Parma,

Via Volturno, 39, 43125, Parma, Italy.

[2] Department of Chemistry, Life Sciences and Environmental Sustainability, University of Parma, Parco Area delle Scienze, 11/A, 43124, Parma, Italy

*, corresponding author, robertin@unipr.it

Phone: +39 0521 903890

Fax: +39 0521 903890

Supplementary Information

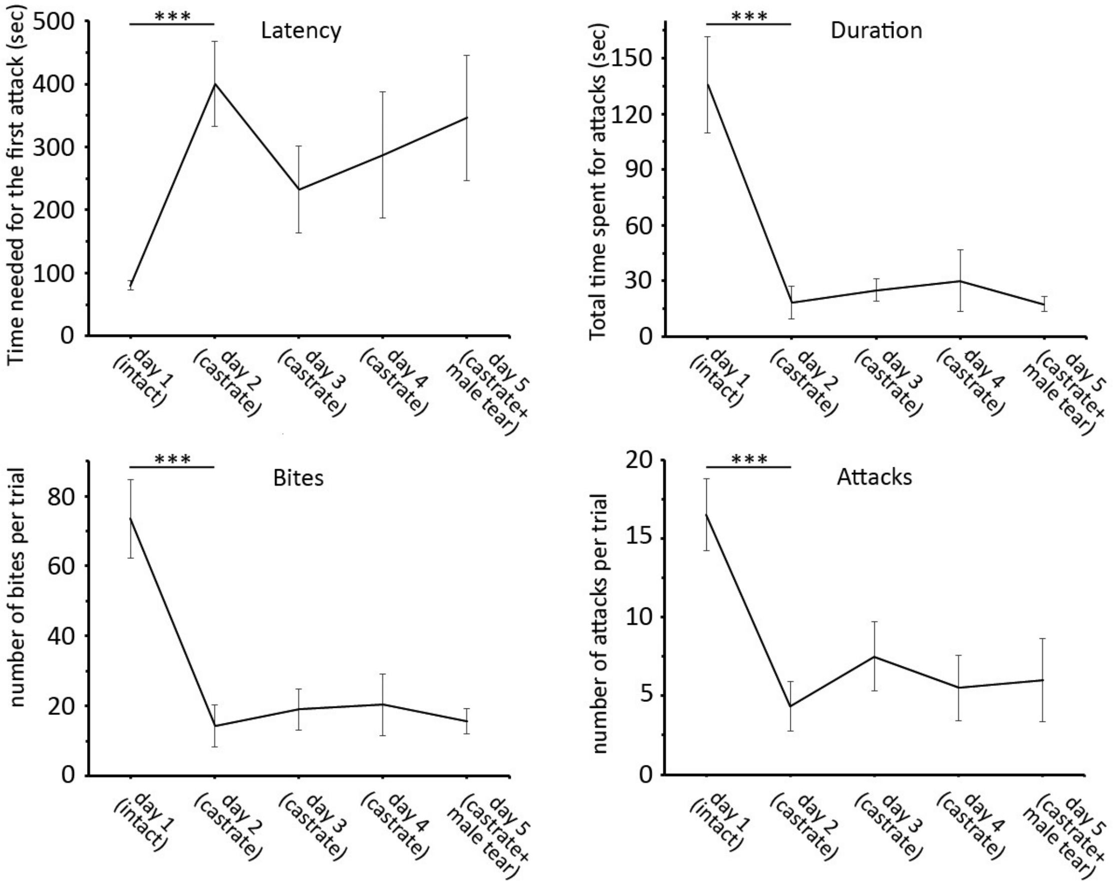

Supplementary Figure S1. Male tear fluid rubbed onto castrated intruder does not affect the aggressive behavior in the resident. Each isolated male mouse was subjected to a 5-day trial. At day 1, the resident mouse was confronted with an adult mouse. At days 2-4, the resident was confronted with a castrated mouse and, finally, at day 5, the resident was challenged with the same castrated mouse rubbed with female tear fluid. Mean ± SE; n=7; ***< 0.001, ANOVA repeated measures.

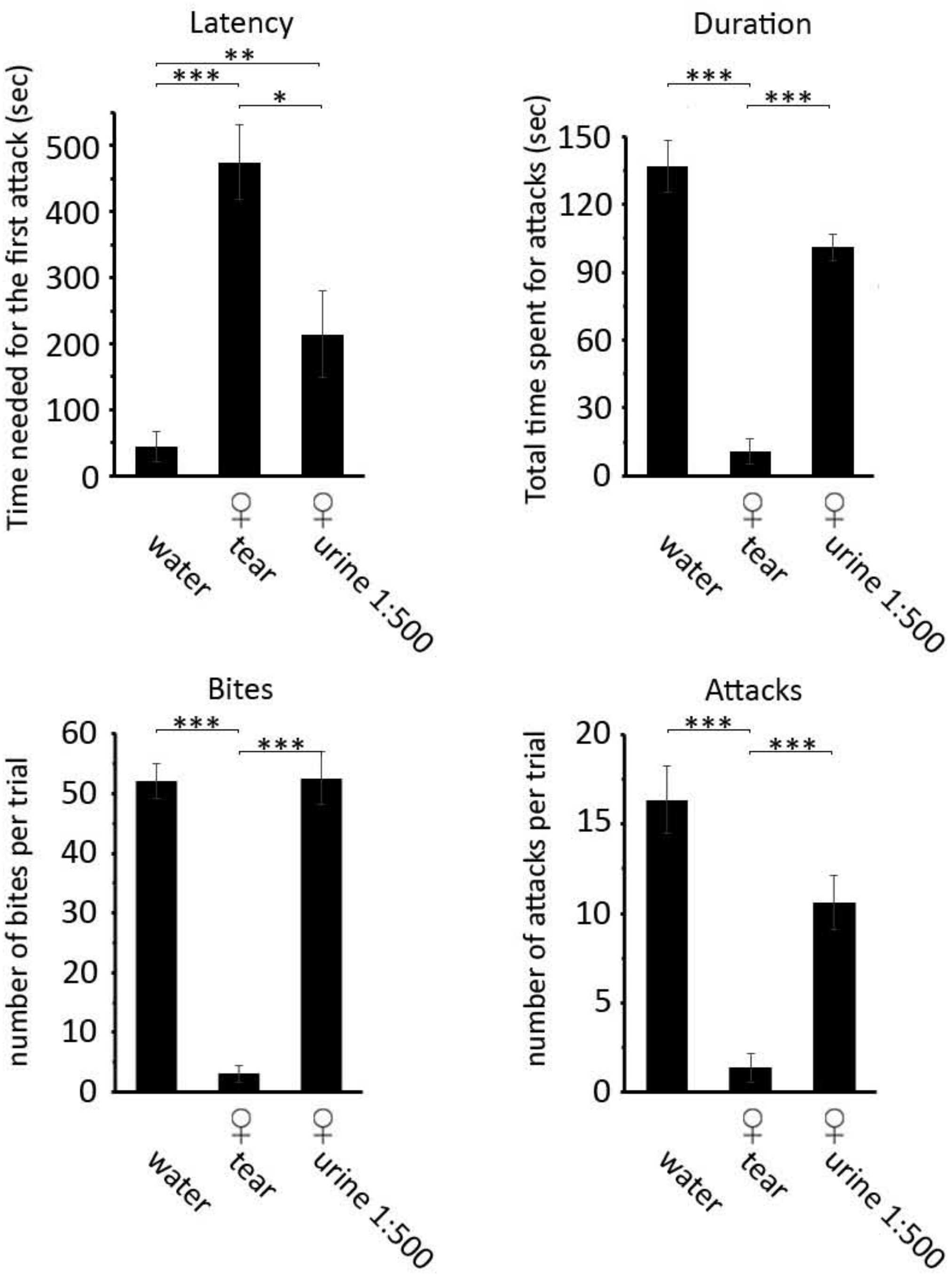

Supplementary Figure S2. Diluted urine is significantly less effective than female tear fluid in inhibiting male aggressive behavior. The value of control column (water) is imported from Fig. 1. Mean ± SE; n=5; *< 0.05, ***< 0.001; unpaired *t*-test.

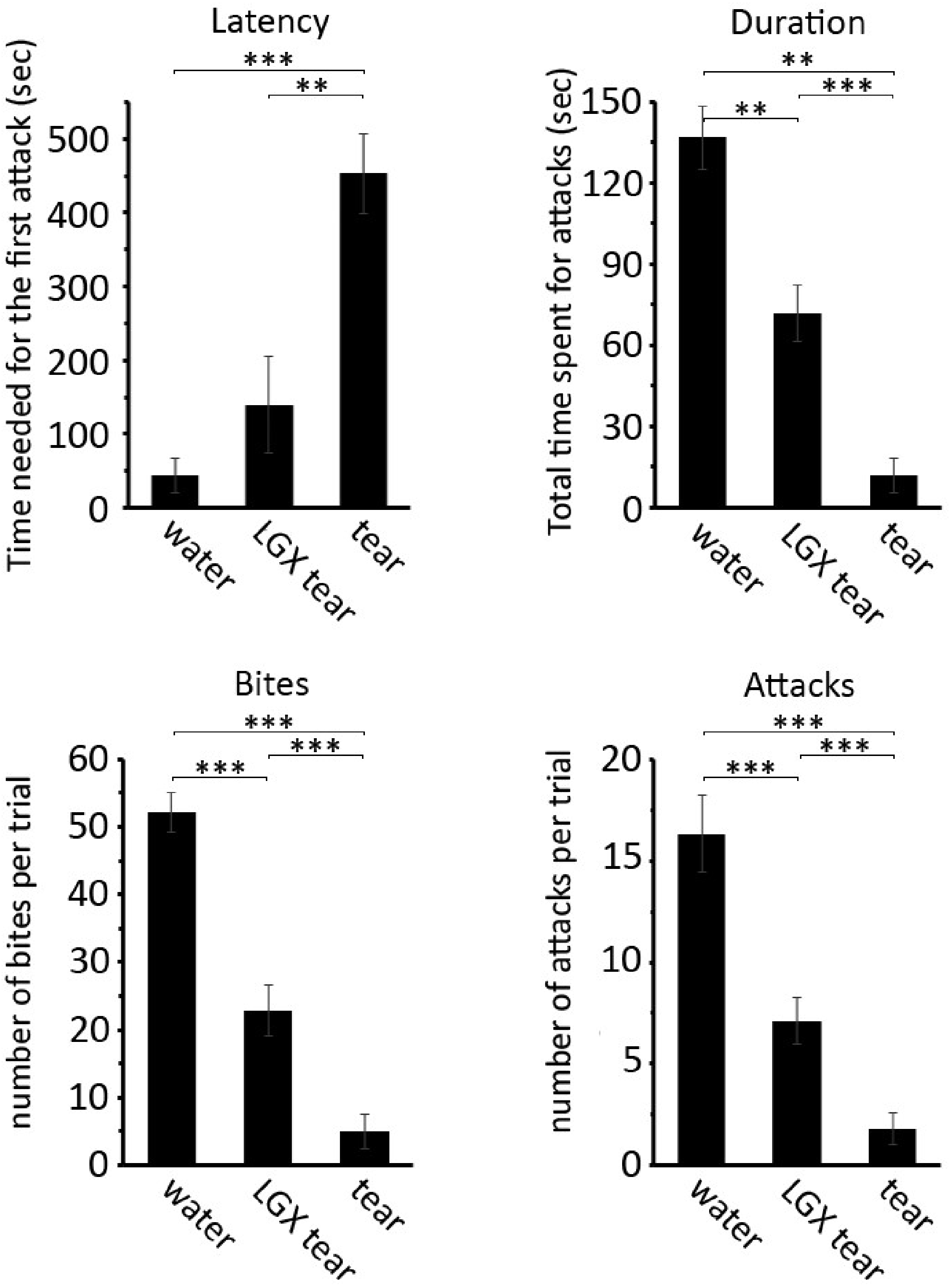

Supplementary Figure S3. Tear fluid obtained from Balb/c females that were deprived of the extraorbital glands is significantly less effective in inhibiting male aggressive behavior. Quantitative analysis of parameters related to aggressive behavior of resident CD1 mice rubbed with tears of normal females and females that underwent surgery for the removal of the extraorbital lacrimal glands (LGX). Values of control column (water) and female Balb/c tear are imported from Fig. 1. Mean ± SE; n=7; **< 0.01, ***< 0.001; unpaired *t*-test.

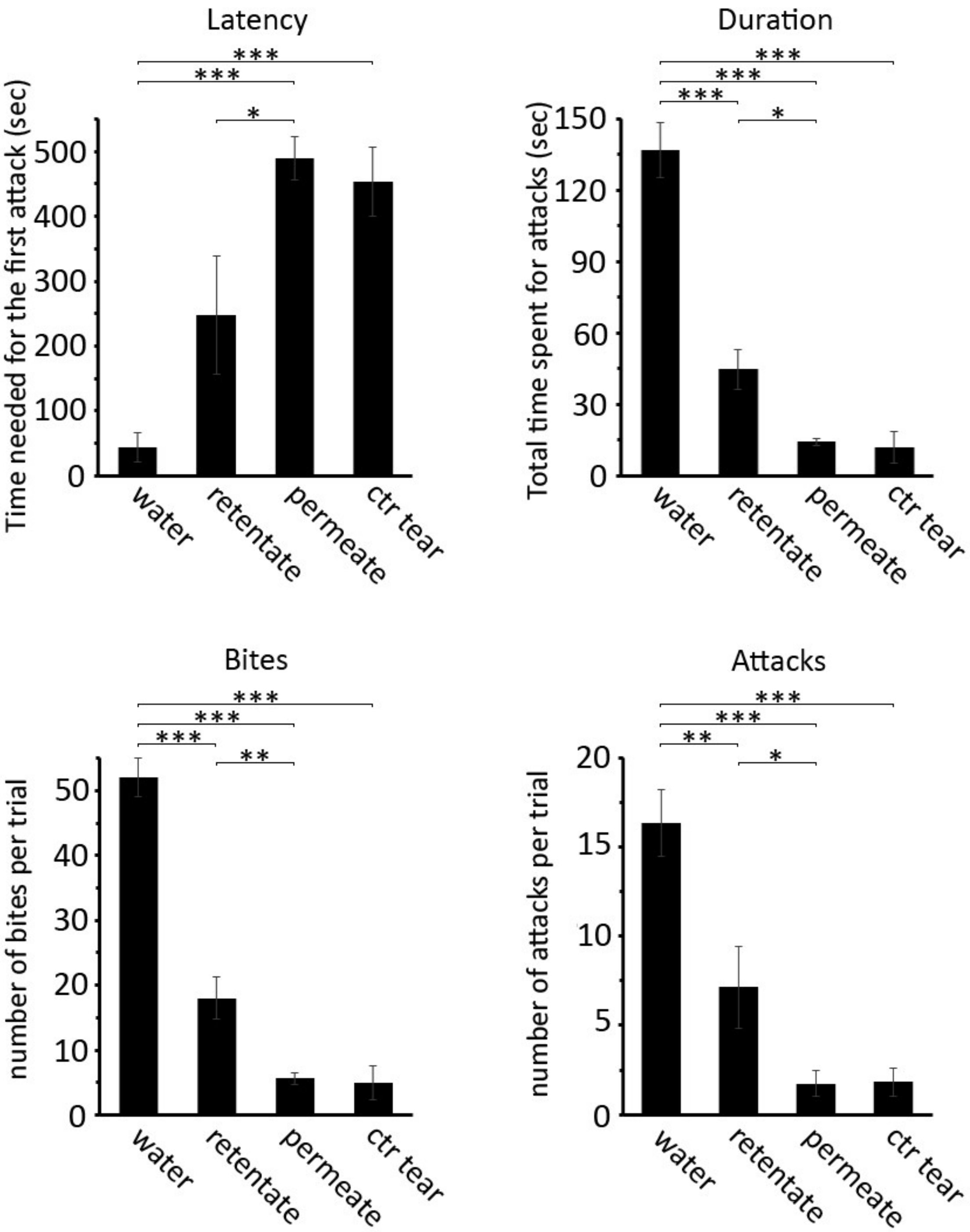

Supplementary Figure S4. The aggression inhibiting pheromone of female tear fluid has a low molecular weight. Quantitative analysis of parameters related to aggressive behavior of resident CD1 mice rubbed with retentate (inside) or permeate (outside) tear fluid following dialysis (membrane cutoff 2KDa). Values of control column (water) and female Balb/c tear are imported from Fig. 1. Mean ± SE; n=7; *< 0.05, **< 0.01, ***< 0.001; unpaired *t*-test.

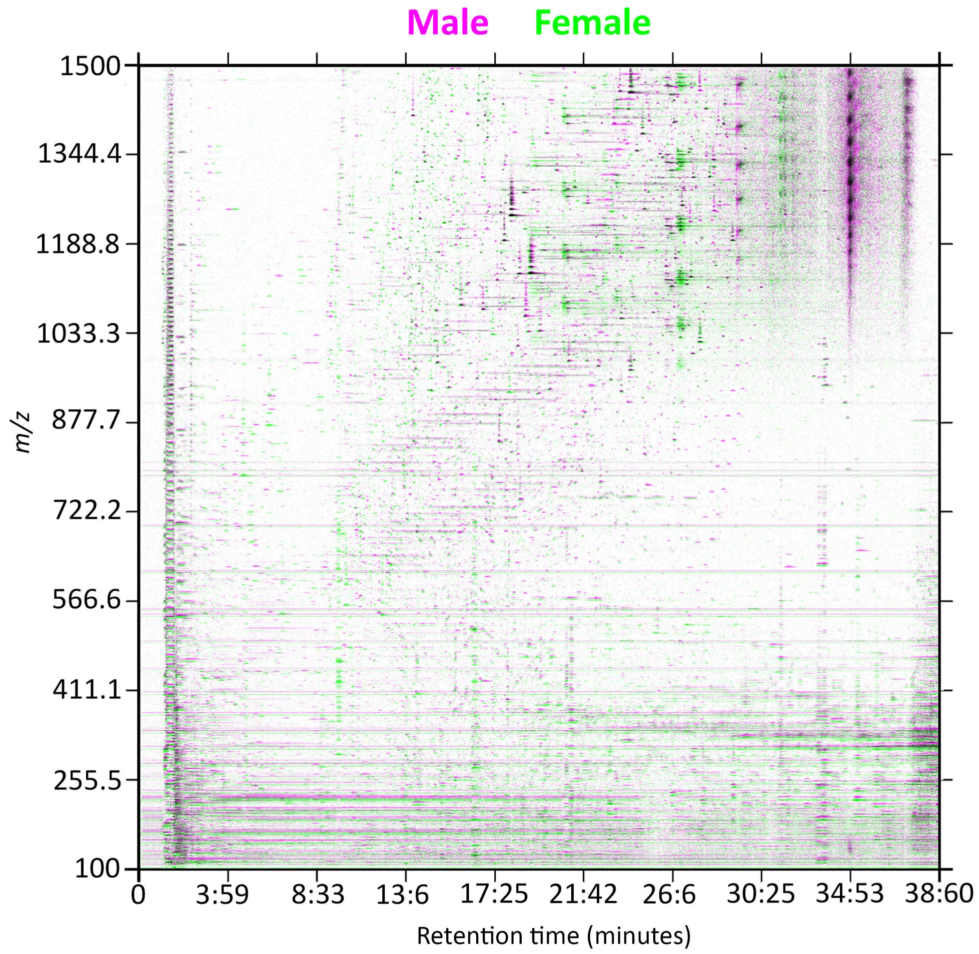

Supplementary Figure S5. MS metabolite profile of male and female tear samples. Ion count as a function of retention time and *m/z* from high-resolution LC-MS experiments acquired in negative mode. Results are from two pooled tear samples from female and male Balb/c mice. Maps are overlaid in different colors for female (green) and male (magenta); their overlap renders as black, indicating peaks that are present in both samples at similar retention time. Color intensity is proportional to the Log of the ion count.
